# Bullous Pemphigoid Onset Following Vaccination in Infancy: A Case Report

**DOI:** 10.7759/cureus.76017

**Published:** 2024-12-19

**Authors:** Sondos Hassanin, Arnav Katira, Nicola Hardcastle

**Affiliations:** 1 Dermatology, Sheffield Children's Hospital, Sheffield, GBR

**Keywords:** autoimmune blistering disease, bullous pemphigoid, infancy, vaccination, vaccine adverse events

## Abstract

Bullous pemphigoid (BP) is a rare autoimmune blistering disease characterised by autoantibodies against basal skin membrane antigens. Although the condition's aetiology remains unclear, recent cases have raised suspicions of an association with immunisation. In this study, we present a case of BP onset in a four-month-old infant following routine vaccination. The infant with BP responded well to systemic therapy. This case emphasises the requirement for further investigation into the potential link between BP and vaccination.

## Introduction

The first case of bullous pemphigoid (BP) was documented in 1970; however, the first reported case of BP in an infant was in 1977, and the cause of the illness remains unknown [1]. Childhood BP demonstrates two peaks in incidence: in the first year of life (infantile BP) and around eight years of age. It is characterised by circulating autoantibodies that attack the basal skin membrane. An increasing number of cases recently intensified suspicions of an immunisation link [2-7].

## Case presentation

We present the case of a four-month-old male infant born via an uncomplicated vaginal delivery at 39 weeks and five days with no complications. The patient was exclusively breastfed. His parents were not consanguineous, and no relevant family history of dermatological conditions existed. The patient underwent routine immunisation in the UK at eight weeks, including DPT (diphtheria-tetanus-pertussis), polio, hepatitis B, *Haemophilus influenzae* type b, whooping cough, rotavirus, MenB (meningococcal group B), and pneumococcal, and at 12 weeks of age (DPT, polio, hepatitis B, *Haemophilus influenzae* type b, whooping cough, rotavirus, and pneumococcal vaccines).

**Figure 1:**
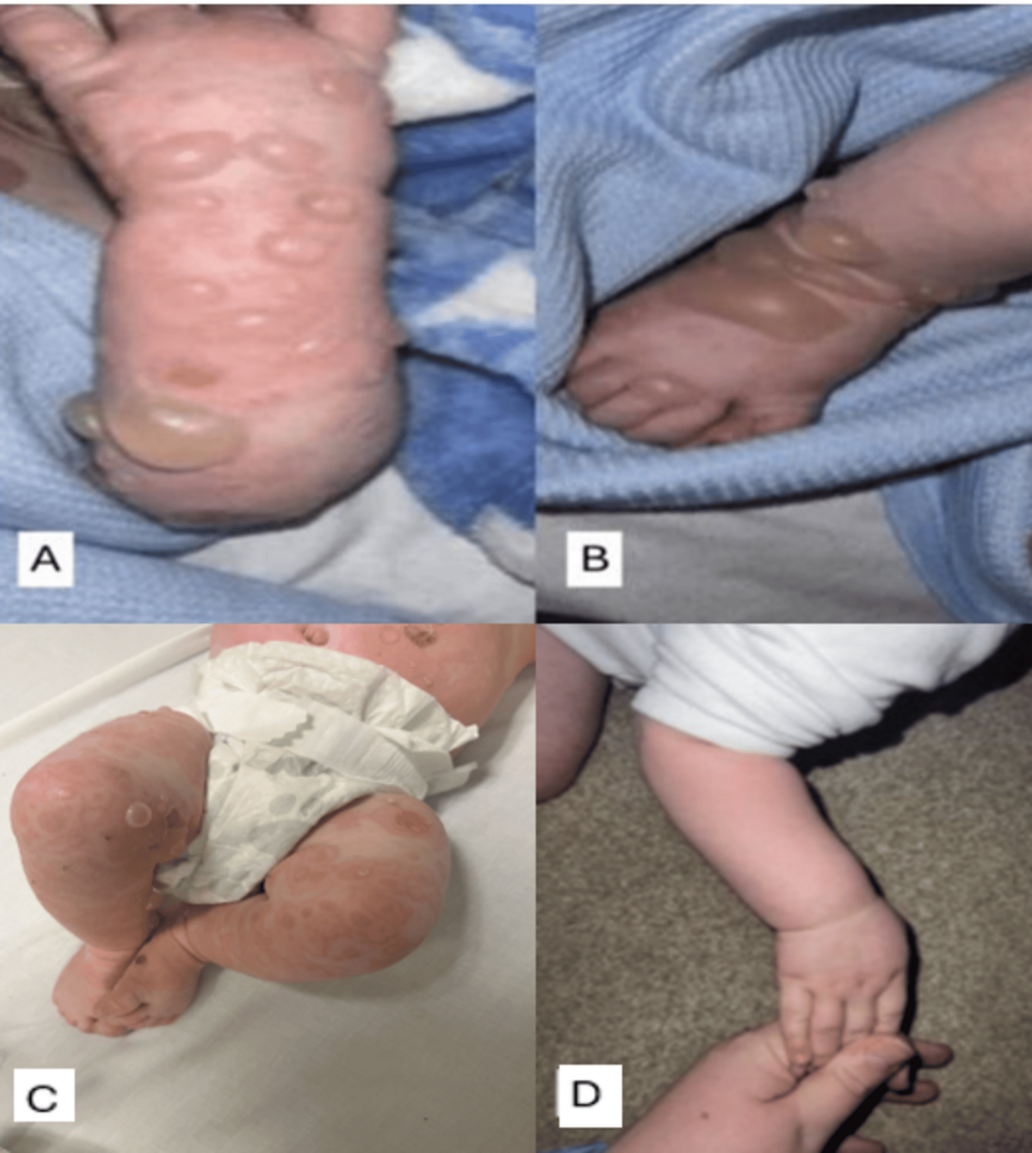
Tense bullae were observed on the (A) hands and (B) feet accompanied by urticarial plaques on the (C) trunk. (D) Clear skin post-treatment.

Two weeks before the eruption, he had been in contact with a dog and had been administered flucloxacillin for seven days. Four days following the 12-week vaccination period, the patient developed an erythematous and oedematous macular rash on the lower abdomen around the umbilicus. Subsequently, these patches began to blister. Chickenpox was initially diagnosed in primary care. However, three days later, the rash eruption progressed to tense blisters developing on the acral sites and eventually spreading to the trunk (Figures 1A, 1B), sparing the back with only annular erythematous patches (Figure 1C). No evidence of mucosal involvement was observed. The initial considerations of the GP (general practitioner) and DGH (District General Hospital) were focused on chickenpox. After the diagnosis of chickenpox, a biopsy was performed, and the patient was given intravenous co-amoxiclav, acyclovir, and paracetamol. The intravenous antibiotics were changed to oral clarithromycin and intravenous teicoplanin to avoid penicillin; however, acyclovir continued.

Table 1 presents the results of the patient's laboratory investigations. Immunological analysis revealed a weakly positive result for anti-BP180 antibodies (IgG), with a value of 2.10 U/mL (reference range: 2.4-8.8 U/mL), which, while lower than the reference range for positivity, still suggested an association with BP in conjunction with clinical findings.

**Table 1:** Laboratory results of the patient CBC: complete blood count; WBC: white blood cells; CRP: C-reactive protein

Parameter	Results	Reference Range
CBC	109 g/L	111–141 g/L
WBC	29.74 × 10^9^/L	6–18 × 10^9^/L
Platelets	483 × 10^9^/L	200–550 × 10^9^/L
CRP	<7 mg/L	<5 mg/L
B-lymphocyte antigen CD19	3.368 × 10^9 ^cells/L	0.12–0.64 × 10⁹ cells/L
Anti-BP180 antibodies (IgG)	2.10 U/mL	2.4–8.8 U/mL

**Figure 2:**
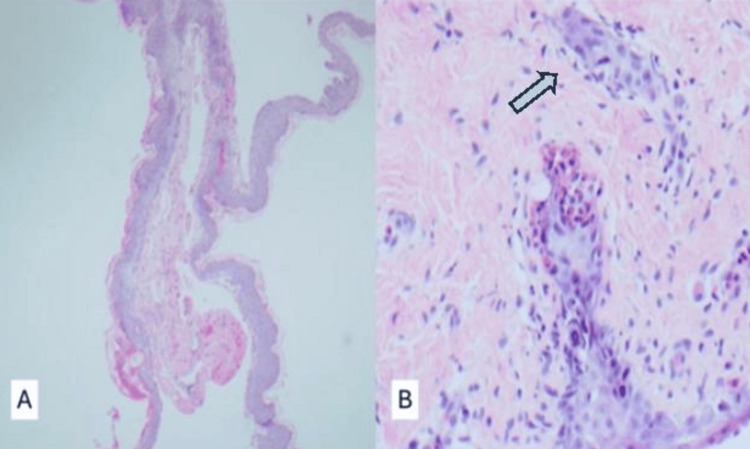
Histological images of (A) low power of sloughed squamous epithelium. The arrow in (B) indicates eosinophils at the dermo-epidermal junction.

Negative results were obtained from virological screening. The microbiological results revealed that all the skin swabs were negative. Histological examination revealed subepidermal blistering with predominant eosinophils and patchy perivascular lymphocytic infiltration (Figures 2A, 2B). Direct immunofluorescence revealed a linear basement membrane zone staining for immunoglobulin G (IgG) and complement C3 (C3), with negative IgA and IgM levels. Enzyme-linked immune sorbent assay (ELISA) detected positive mitotic spindles, epidermal basement membranes, and anti-bullous pemphigoid 180 (anti-BP180) antibodies. Therefore, the patient was diagnosed with BP.

Treatment was initiated with prednisolone 6 mg (1 mg/kg) because of the unsatisfactory response, and the dose was increased to 12 mg (2 mg/kg). A potent topical steroid, clobetasol propionate, was applied to the new blisters. Steroids were increased after 48 hours, and mycophenolate mofetil (MMF) was introduced simultaneously. The dose of steroids was increased on day two of hospitalisation due to the patient developing neurological symptoms. Subsequently, MMF was introduced, and the patient was administered a single dose of MMF 60 mg while waiting for approval for intravenous immunoglobulin (IVIG) because of active blistering, an unsatisfactory response, and concerns about side effects. MMF was initiated on the sixth day of hospitalisation; a single dose of 60 mg was given. Two doses of IVIG (6 g, 1 g/kg over two days) were administered, reducing the existing inflammation and the new eruption. Disease control has progressed well. Erythromycin 120 mg twice daily (40 mg/kg/day) was initiated to prevent further recurrence and was continued alongside a weaning course of prednisolone.

Antibiotics, such as flucloxacillin, were given prehospital for seven days. Others were given in DGH for less than 24 hours, pending transport. The blistering cleared off, and erythromycin was stopped after three months without reoccurrence of the condition. The supportive care included solutions, intravenous fluids to treat dehydration or when there is an imbalance of electrolyte content in the blood, the use of antipyretics in cases of fever and analgesics where the patient is in pain, the provision of nutrition where necessary throughout the course of the patient's treatment and recovery, and monitoring the patient's vital signs and biochemical parameters in order to estimate the condition's activity and treatment efficacy.

## Discussion

Childhood BP is rare, with two reported peaks in incidence: the first year of life (infantile BP) and around eight years of age. It occurs slightly more frequently in females. BP is characterised by circulating autoantibodies that target basal skin membrane antigens BP180 and BP230. The aetiology of BP is poorly understood, but risk factors include old age, neurological conditions, and specific medications, such as loop diuretics and neuroleptics. Disease progression is thought to be better in young patients, as it tends to be more localised and has a lower chance of recurrence. Additionally, mucosal involvement is rarely reported, possibly due to the pattern of circulating antibodies. Despite increasing reports of BP following vaccination, little evidence has established a profound relationship. The timeline from vaccine administration to clinical presentation is approximately five to seven days. An unanswered question is whether routine vaccinations trigger autoantigens against the basal membrane, causing subclinical BP in genetically predisposed individuals. The simple detection of type IV collagen in paraffin-embedded skin biopsies has been used to differentiate between BP and other subepidermal bullous diseases [8].

The mainstay of treatment for BP is systemic corticosteroid therapy, but managing the extent of the disease and its adverse effects is crucial. Topical steroids are used for limited cases. Oral anti-inflammatory antibiotics, such as tetracyclines, have been successfully employed, although age-specific antibiotics are necessary because doxycycline is contraindicated in patients under the age of eight years [9]. In many cases, systemic therapies, including MMF, azathioprine, methotrexate, cyclophosphamide, IVIG, rituximab, and omalizumab, may also be used [10]. In our case, the patient received a 12-week immunisation approximately three to four days post-seven-day flucloxacillin course and developed an eruption after four days of immunisation. Despite the fact that there may be a temporal relationship between vaccination and BP, we were unable to conclude that it has a causal relationship. It may also occur due to immune modulation after frequent vaccination, or it could be coincidental [6].

Future research should focus on analysing the routes through which vaccinations modulate the immune system since these may increase the risk of autoimmune diseases, such as high blood pressure, in susceptible individuals. It is essential to comprehend the environmental and genetic elements that may increase the likelihood of these adverse consequences. A national post-vaccination data registry must also be created in order to rigorously monitor and evaluate rare vaccine-related side effects, such as blood pressure. This kind of registry would improve our ability to monitor long-term health outcomes and provide academic and medical professionals with valuable information that would enable them to identify vaccination-related risks and anomalies. This proactive approach will also ensure early discovery and treatment of rare challenges, enhancing the safety and efficacy of vaccination programs globally.

## Conclusions

In conclusion, we reported a patient who developed BP after receiving regular vaccinations. This patient was effectively treated with a combination of IVIG, corticosteroids, antibiotics, and a single dose of MMF. Since BP is an uncommon condition in newborns and is not well understood by paediatricians and dermatologists, differential diagnosis is necessary, particularly when blisters or eruptions arise after vaccination in infants. In order to manage complex autoimmune disorders and ensure quick symptom resolution and recovery, this example emphasises the significance of a thorough and customised treatment approach.

## References

[1] Barreau M, Stefan A, Brouard J, Leconte C, Morice C, Comoz F, Verneuil L (2012). Infantile bullous pemphigoid (Article in French). Ann Dermatol Venereol.

[2] Rajaram Mohan K, Govindasamy Sugumar SP, Fenn SM, Pethagounder Thangavelu R (2023). Recurrent bullous pemphigoid: a case report and literature review. World Acad Sci J.

[3] Baroero L, Coppo P, Bertolino L, Maccario S, Savino F (2017). Three case reports of post immunization and post viral bullous pemphigoid: looking for the right trigger. BMC Pediatr.

[4] Ferreira BR, Vaz AS, Ramos L, Reis JP, Gonçalo M (2017). Bullous pemphigoid of infancy - report and review of infantile and pediatric bullous pemphigoid. Dermatol Online J.

[5] Bisherwal K, Pandhi D, Singal A, Sharma S (2016). Infantile bullous pemphigoid following vaccination. Indian Pediatr.

[6] Hafiji J, Bhogal B, Rytina E, Burrows NP (2010). Bullous pemphigoid in infancy developing after the first vaccination. Clin Exp Dermatol.

[7] Martinez-De Pablo MI, González-Enseñat MA, Vicente A, Gilaberte M, Mascaró JM Jr (2007). Childhood bullous pemphigoid: clinical and immunological findings in a series of 4 cases. Arch Dermatol.

[8] Reis-Filho EG, Silva Tde A, Aguirre LH, Reis CM (2013). Bullous pemphigoid in a 3-month-old infant: case report and literature review of this dermatosis in childhood. An Bras Dermatol.

[9] Williams HC, Wojnarowska F, Kirtschig G (2017). Doxycycline versus prednisolone as an initial treatment strategy for bullous pemphigoid: a pragmatic, non-inferiority, randomised controlled trial. Lancet.

[10] Neri I, Evangelista V, Guglielmo A, Sechi A, Virdi A (2021). A case of bullous rash apparently triggered by meningococcal and rotavirus vaccines in an infant: focus on infantile bullous pemphigoid. Dermatopathology (Basel).

